# Retrospect of the Medical Sciences

**Published:** 1843-05-13

**Authors:** 


### DISEASE OF MITRAL VALVES.

Mr. Adams communicated a case of disease of the mitral valves. The subject was a gentleman temperate in his habits, but who had formerly suffered repeated syphilitic attacks, for which he had undergone several mercurial courses. About six years ago he began to feel a difficulty of breathing, and afterwards hæmoptysis and palpitations. Three months ago (on the 3rd of September), while turning in his bed, he perceived a remarkable irregularity in the action of the heart. A few weeks back he came to Dublin, and applied for advice to Mr. Adams, who, on examining him, found that his limbs were anasarcous, and there were evident symptoms of a disease of the heart, but it was doubtful whether it were aneurism or disease of the valves. The second sound of the heart was accompanied by a sound like that observed in cases of aneurism. A consultation was then held with Dr. Stokes, who was of opinion that the mitral valves were diseased, and that the death would be very sudden. The treatment adopted consisted in the exhibition of small doses of calomel, squill, and digitalis, together with small bleedings by leeches, which were used in preference to small bleedings from the arm, in consequence of the patient's statement that venesection caused him to faint, and this is objectionable in diseases of the heart. At this time, although the action of the heart was violent, the pulse at the wrist was very weak, the respiration was generally sound, but at night dyspnœa occurred in paroxysms. The death was very sudden; it occurred soon after breakfast, while the patient was on the night-chair. The left ventricle was found to be considerably dilated, and the mitral valves indurated, thickened, and infiltrated with a yellowish material, which, in some parts, was ossified. The appearances were well delineated in a drawing produced by Mr. Adams.—*Dub. Med. Journ.*

### TANNIN IN HOOPING COUGH.

M. Sebregondi has found decided benefit from the employment of pure tannin in the asthenic stage of hooping cough. He has administered it in doses of a quarter to half a grain every two hours in conjunction with sedatives, as extract of hemlock, &c., and purgatives, as infusion of senna, under which treatment the paroxysms have entirely ceased in a short time. Any constipating or griping effect of the remedy is corrected by its combination with the above medicines. With respect to the hemlock, it may be added that Dr. Meyer, of Kreutzburg, has found tannin as obtained in infusion of galls an efficient remedy against the poisonous effect of this substance, emetics of sulphate of zinc having been previously employed to empty the stomach.—*Lon. Lancet, from Med. Zeit.*, No. 50.

### OINTMENT OF THE EXTRACT OF NUTGALLS.

Mr. Daniel S. Jones, a recent graduate of the Philadelphia College of Pharmacy, states that when nutgalls are treated with water by the displacement process, they yield 63 per cent. of dry extract, about two-thirds of their weight, and proposes an ointment formed by triturating two scruples of the extract of galls with a little water, and afterwards with seven drams of lard as a substitute for the officinal ointment made with ordinary powdered galls.

When thus made, this ointment embodies all the activity of the nutgalls, without that gritty, uneven character of the ordinary ointment, due to the uneven division of the powder, and which amounts to an objection to its use in some cases of extremely irritable hemorrhoids.

The ointment possessed a perfectly uniform consistence, but was objected to as being too soft. Simple cerate was proposed as a substitute for lard. In the ordinary ointment, the dry bulky nature of the powdered galls compensates for the softness of the lard. In other respects the proposed ointment was approved of as an efficient and eligible preparation.—*Am. Journ. of Pharm.* April, 1843.

---

### THE INDIAN HEMP.

At a meeting of the Medico-Botanical Society of London, March 22d, a communication from Mr. Ley was read detailing the results of his experience with the Cannabis Indica, or Indian hemp, in the treatment of certain convulsive and inflammatory diseases. One of the most important and interesting of the cases detailed by him was that of a lady who had been confined to the hydrostatic bed for five years from disease of the spine and hip, and in whom, whenever she was removed from the bed for the renewal of the India-rubber sheet covering it, there was produced a disturbance, little felt at the time, but inducing at night a succession of violent spasms of the muscles of the spine, drawing the body back into the form of an arch, and as suddenly relaxing. These spasms would follow each other rapidly through the night, producing faintness, sickness, and insensibility, all through the next day. This alternate state of nocturnal tetanus and daily fainting continued, generally, after each moving, for a fortnight or three weeks, obstinately resisting every medicine that could be applied for its relief. When Mr. Ley obtained possession of some of the resinous extract of hemp, he had pills of one grain weight made, of which he gave his patient six, directing her to take one on the occurrence of the first symptom of the spasm, and to repeat the dose every half hour until the spasm was relieved, or some other reason offered for their discontinuance. Five pills were taken, when she felt overpowered, the muscles relaxed, and she fell into a profoundly tranquil sleep, likened by her afterwards to a trance, because she was for a long time after she had apparently fallen asleep conscious of passing events, but unable to make known her perceptions. The spasmodic attacks returned for several successive nights, but were not so violent nor of such duration as previously. The opinion of this lady, who, from long illness, is familiarised with medicines, is, that the extract of hemp affects the muscles principally, relieves ordinary pains less surely than opium, disturbs the stomach little, if at all, but produces an unpleasant sensation in the head, not excitement, but objects appear before the eyes which do not really exist; thus she may see a book so plainly on her bed as to reach out her hand to take it, or she may seem to be holding a newspaper. Mr. Ley has further found the resinous extract of hemp of service in the treatment of acute rheumatism, cholera, chorea, effusion into the knee-joint, housemaid's-knee, enlarged ganglia, &c.—*London Lancet*, April 1, 1843.

---

### CONCLUSION OF MM. DANGER AND FLANDIN'S RESEARCHES CONCERNING THE EFFECTS OF ARSENIC ON WOOL-COVERED ANIMALS, &c.

1. The sheep that survived after taking four drachms of arsenious acid, having been killed on the thirty-eighth day after, no part of the carcase was found, on the autopsy, to contain a single appreciable trace of arsenic. A dog to which the viscera were given to eat exhibited not the slightest sign of illness, nor could arsenic be detected on analysis either of its fæces or urine; and six persons who partook of the muscular fibre as food lived on it for twelve days without feeling any inconvenience or symptom distinguishing it from meat of other descriptions.

2. The dog that ate the viscera of three poisoned sheep did not experience fatal results, and when killed on the ninth day from the reception of this food, exhibited a healthy internal appearance, without any trace of arsenic whatever, the entire poison having passed off in the urine during the six days immediately following the introduction of the poison into the system. The comparative harmlessness of the poison on the dog, as compared with the sheep, may be accounted for from the far smaller extent (about one fifth) of the intestinal canal, as well as the much greater muscularity of the tissues connected with the digestive organs of the carnivorous animals: these causes render the digestion, absorption, and the secretions generally much more active in the dog than the sheep.—*Ibid, from Gazette des Hôpitaux.*

---

### NAPHTHALINE.

This substance has been employed with success by M. Emery in the treatment of psoriasis and lepra vulgaris, twelve cases out of fourteen in which it was tried having been entirely cured by its use. M. Emery recommends its application to the skin in the form of an ointment composed of from one to two drachms of naphthaline to an ounce of lard. Considerable irritation is sometimes produced; but this seems to be readily allayed by emollient affusion and cataplasms, and the remedy usually effects a cure in a few weeks or months. No particular course of diet is enjoined.—*Ibid, from L'Experience.*

---

### TREATMENT OF SALIVATION.

To combat mercurial salivation, I prefer applying acid. hydrochlor. fort. to the gums and tongue when they are ulceraed, repeating the application every day, or every other day. The bleeding of the surface ought not to be an obstacle. The acute pain it produces soon ceases, and nothing equals its beneficial effects. Of course the peculiar indications which may present themselves must not be neglected.—*M. Ricord on Venereal.*

